# Growing up and Role Modeling: A Theory in Iranian Nursing Students’ Education

**DOI:** 10.5539/gjhs.v7n2p273

**Published:** 2014-11-16

**Authors:** Jamileh Mokhtari Nouri, Abbas Ebadi, Fatemeh Alhani, Nahid Rejeh

**Affiliations:** 1 Behavioral Sciences Research Center (BSRC), Faculty of nursing, Baqiyatallah University of Medical Sciences, Tehran, Iran; 2 Nursing Department, Medical Sciences Faculty, Tarbiat Modares University, Tehran, Iran; 3 Elderly Care Research Center, Department of Nursing, Faculty of Nursing and Midwifery, Shahed University, Tehran, Iran

**Keywords:** education, grounded theory, Growing up and Role Modeling Theory, growth, nursing, role modeling

## Abstract

One of the key strategies in students’ learning is being affected by models. Understanding the role-modeling process in education will help to make greater use of this training strategy. The aim of this grounded theory study was to explore Iranian nursing students and instructors’ experiences about role modeling process. Data was analyzed by Glaserian’s Grounded Theory methodology through semi-structured interviews with 7 faculty members, 2 nursing students; the three focus group discussions with 20 nursing students based on purposive and theoretical sampling was done for explaining role modeling process from four nursing faculties in Tehran. Through basic coding, an effort to comprehensive growth and excellence was made with the basic social process consisting the core category and through selective coding three phases were identified as: realizing and exposure to inadequate human and professional growth, facilitating human and professional growth and evolution. The role modeling process is taking place unconscious, involuntary, dynamic and with positive progressive process in order to facilitate overall growth in nursing student. Accordingly, the design and implementation of the designed model can be used to make this unconscious to conscious, active and voluntarily processes a process to help education administrators of nursing colleges and supra organization to prevent threats to human and professional in nursing students’ education and promote nursing students’ growth.

## 1. Introduction

The main goal of nursing education is to train qualified and competent nurses who are knowledgeable and skillful enough to maintain and improve the quality of care provided for the patients (Hossein et al., 2010). In other words, nursing education basically focuses on the transmission of knowledge to students (as the future nurses) and assists them to acquire the necessary skills and attitudes to be competent clinical nurses (Salsali, 2005). Moreover, nurses’ role goes beyond education and evaluation of quality of nursing care is emphasized (Istomina et al., 2011).

Studies conducted in Iran on nursing education indicate the inefficiency of clinical and theoretical trainings for nursing students (Rejeh et al., 2011), which has resulted in a low quality care and also a theory and clinic gap (Vaismoradi et al., 2011). Many Iranian students experience anxiety as a result of feeling incompetent in terms of clinical skills and theoretical knowledge for meeting patients’ various needs (Cheraghi et al., 2010). It should be noted that many teaching methods and strategies have been devised to meet students’ educational needs among which role modeling is the newest one (Karimi Moonaghi et al., 2009).

In Iran, nursing trainers believe that role modeling teaching method is the most effective approach for developing the experiences and professional attitudes of students during clinical situations (Hossein et al., 2010). The term ‘role model’ coined by Merton refers to the person who sets a positive example and is worthy of imitation (Perry, 2009). Role modeling focuses on the fact that trainees would try to imitate their trainers’ behavior, because of their respect for and trust in the mentors (Weng et al., 2010).

A mentor is a role model who inspires, a guide who encourages, a counselor, teacher, advocate, and an advisor (Kuwabara & Johnson, 2009). Role models not only teach professional thinking, behaviors, and attitudes, but also facilitate the development of learner’s beliefs and practices, which assures the future provision of care quality (Perry, 2009). The development of students’ competence and confidence in nursing discipline is believed to be under the tremendous influence of a good role model (Fluit et al., 2011).

Being a good role model is a sign of instructor’s competency, which facilitates teaching theoretical and clinical aspects (Wolf et al., 2009). To advance nursing profession, nursing educators should be acceptable role models (Klunklin et al., 2011). However, the reason for the importance of role modeling is seldom discussed. In an era in which ‘learning targets’, ‘learning moments’ and ‘transfering mechanisms’ are emphasized, the function of role models in clinical situation should also be given more attention. Therefore, conducting studies help with providing a clearer picture of the function and the value of role models in nursing education and practice (Stegeman, 2001).

Ericksin, Tomlin & Swains’ Modeling and Role-Modeling theory was presented in 1982. Role-Modeling is the facilitation of an individual in attaining, maintaining, or promoting health through purposeful intervention. Modeling is defined as “the process a nurse uses as she/he develops an image and understanding of the person’s world within the person’s perspective”. Modeling is a central concept because understanding the client’s viewpoint is the foundation for implementing the nursing process. Role-Modeling can occur only after the nurse accurately understands the client’s worldview. Modeling and Role-Modeling is a client-centered nursing theory that places the client’s perceptions, or model of world at the center of the nurse-client interaction. Modeling and Role-Modeling serves as a foundation for nursing education (Schultz & Peterson, 2004).

According to Hossein et al’s study (2010) in Iran, nursing trainers believe that role-modeling teaching method is the most effective and accurate approach to transfer the experience and professional attitude to students in clinical training (Hussein et al., 2010). Despite the importance of role modeling and the presence of qualified model nursing instructors, a few studies have been carried out in Iran on role modeling and strategies to incorporate it into nursing education from the perspectives of nursing instructors and nursing students and role modeling process is not well known.

Therefore, this study aimed to explore the perspectives and experiences of Iranian nursing students and instructors about role modeling and devise strategies to incorporate role modeling into nursing education.

## 2. Methods

### 2.1 Data Analysis

In the first phase, Glaserian’s Grounded theory method was applied in order to explain the role modeling process (Glaser, 1998). All participants’ statements typed in the Text Writing Office software (Word) and then transferred to MAX Q-DA analysis (version 2007) and then all of data was analyzed. Data analysis methods included two steps of coding, basic coding and selective coding through repeated line by line reading of transcripts and memos.

### 2.2 Data Collection and Interview Guides

Data collection was conducted by one of the researchers who did qualitative research for her doctoral dissertation. The collection was performed by focus group discussions and in-dept interviews from November 2010 to October 2011 in Tehran’s nursing schools and continued until data saturation was achieved.

Each session of focus group discussion lasted for 90 to 120 minutes on average. The participations in the groups were homogenous in terms of their educational levels. The sessions were conducted by the first and second authors. The length of each interview ranged from 20 minutes to 120 minutes. Interviews were done in a private setting and transcription was done after each interview. In-depth interviews, semi-structured questions were used for data collection. Questions guides from nursing instructors and students were as follows:

1) Would you please define your professional biography (experiences in classroom education and clinical training?)
2) Would you please describe one of your working days?
3) Explain your classroom management and clinical training?
4) Elaborate your experiences of yours teachers as nursing model?

These questions were designed as the interview guide, and participants’ answers led the interview process. Exploratory questions such as “Explain more” or “What does it mean?” and the like were used to encourage participants to discuss their experiences to deeper insights. Individual semi-structured interviews were conducted in a private classroom at the workplace.

### 2.3 Participants

Three focus-group discussions (FGD) with 20 nursing students and two semi-structured face-to-face interviews with nursing students as well as seven semi-structured interviews with nursing instructors were conducted to gather data.

### 2.4 Ethical Considerations

The study was approved by ethics committee of Baqiyatallah University of Medical Sciences. All the participants were informed about the study method and purpose. They were informed that participation in the study was voluntary and that they could refuse to participate or withdraw from the study at any time. Moreover, the participants were reassured that their responses would be confidential and their identity would not be revealed in research reports. Finally, those who agreed to participate in the study signed a written consent form.

### 2.5 Rigor

The credibility of the data was established with two PhD candidates in nursing as peer checking. The authors coded and categorized the data independently and then their findings were compared. When the authors disagreed, discussions and clarifications continued until to reach a consensus. Moreover, a summary of the interviews was returned to the participants as the member checking and it was confirmed that the researcher was representing their ideas (Graneheim & Lundman, 2004).

## 3. Results

### 3.1 Participants

Students were with the mean age of 26.5±6.27 years. They mostly were (53.8%) bachelor’s degree students and female (66.3%). Seven nursing instructors with the mean age of 45±2.38 years were participated in individual interviews. Five and two of them had PhD and master’s degree in nursing, respectively and six were married.

### 3.2 Results of Data Analysis

Through basic coding, 2510 primary concepts, 57 subcategories and 12 categories were extracted. Effort to comprehensive growth and excellence was made with the basic social process consisting as the core category. To complete the theory, unrelated data around the core category were excluded.

**Figure 1:**
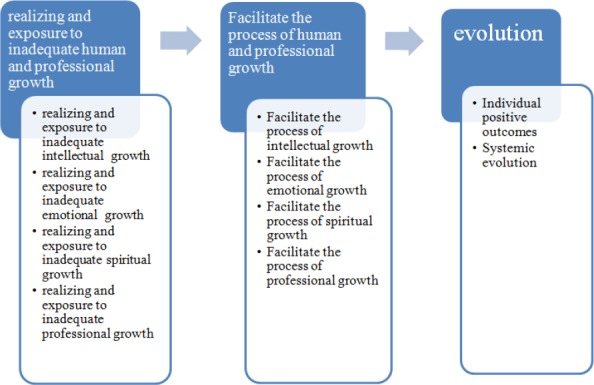
The basic social psychological process of “effort to comprehensive growth and excellence”

After the selective coding, three phases were identified as: realizing and exposure to inadequate human and professional growth, facilitating human and professional development and evolution (Figure 1).

### 3.3 Core Category: Effort to Comprehensive Growth and Excellence

The core category of the experience of nursing instructors and students in Iran was identified as effort to comprehensive growth and excellence. Data analysis showed that participants’ main concern is how to deal with perceived threats from human development and professional abnormalities and they used the basic strategy “effort for comprehensive growth and development” against these concerns to facilitate the comprehensive growth. Effort for growth and development was the most common social psychological process among the data.

As one participant mentioned as follows:

“My role model’s behaviors and was performances behavior & action of my role model was so that students would understand that he loves the students to learn and develop in all fields (FG3).

### 3.4 Process of Role Modeling in Nursing Student’s Education

**First phase: Realizing and exposure to inadequate human and professional growth**

*A:* *realizing and exposure to inadequate human growth*

Participants’ experiences concerning “ineffective interactions”, “low self-esteem”, “low motivation” stated facing and perceiving threat of emotional growth barriers. “Weakness in seeking perfection and being evolved”, “weakness in ethics” and “weakness in spiritualities and beliefs” became substructure of the level of greater content named “perceiving threaten of spiritual growth barriers” and “need to develop thinking” and “need to consultation and guidance” formed content level of “perceiving threaten of rational development barriers”. All the emotional, spiritual and intellectual dimensions are human existence dimensions. Some examples of the participants’ experiences:

“We have a professor that his/her knowledge and also his/her education is good and he/she is dominant on the materials but I feel that this predominate made a pride for him/her that intercepts the students to come and speak.” (FG2)

“Some teachers do not observe ethics, they are rude. We had in the PHD level a person who was scientifically very productive and from responsibility and position point of view he/she was in a high rank but he/she was morally zero. He/she was grumpy.” (S2)

“Some students’ mind was not engaged with class subjects and activities. So I said to myself what conditions can I make in order to engage their mind?… when the mind is not engaged, it does not think, it assembles. I said what can I do in order to understand that their mind has been engaged? So I considered it necessary to have consultation and guidance.” (T3)

B: realizing and exposure to inadequate professional growth

Feeling of professional anomalies threat was due to inefficient education-research system, inappropriate professional position and interprofessional ineffective interactions. In this study inefficient education-research system returned to poor academic performance of professor and student, inappropriate training program, ineffective evaluation system, negative impact of colleagues, feeling of threaten about doing incorrect research and lack of use of the study results. Inappropriate professional position had been stated following negative attitude and approach of the people towards nursing profession and negative attitude of the teachers and nursing students and lack of professional autonomy. In ineffective interprofessional interactions, ineffective interactions with the patient and colleagues were among the points that the participants pointed to them. Four participants testified as follows:

“…it doesn’t matter at all for the system managers that what happen in the system. Top-level managers do not care much about procedure and process, they mostly care about the outcomes, whether the students have been graduated on time or not? But they do not care about the way of teachers’ performance? (T3)

“Many of the researches which have been done in nursing colleges are not used at bedside”. (T6)

“The approach of the people towards the profession always bothers me”. (T5)

“My model teacher said that why when the interns go to the round with their professor, you come out? You must be in the room. You are nursing students; you must have communication with members of treatment team”. (FG2)

**Second phase: Facilitating the process of human and professional growth**

A: Facilitating the process of human growth

Model teachers through “effective communications”, “instilling a sense of positive identity”, “to loving others”, it means through “effective interactions” facilitate emotional growth process. Also in facing and perceiving spiritual growth barriers, with “having religious beliefs” and “adherence to ethics” it means with their own “special character features” helped students’ growth. On the other hand after facing and perceiving intellectual develop barriers with “guiding thought” and “strengthening critical thinking” it means through “managing thought” they attempted to facilitate process of students’ rational growth. Finally strategy to facilitate human development had been done through effective interactions, thought, management and special character features. Some examples of the participants’ experiences:

“He/she respects to the character of counterparty at any level that he/she is.” (FG3)

“I saw that they love themselves and their lives and their nursing field, and this love is in every second of their lives and this had been widespread in a way that it has affected me as a student.” (FG1)

“God observes all our actions, and from the beginning of my teaching work, I promised to God to work in a way that God is satisfied.” (T7)

“It is counseling technique that can survive student’s thought, this surviving of thought, I call it learning.” (T2)

B: Facilitating the process of professional growth

Model teachers after facing professional anomalies used different strategies in order to solve this challenge. In this way that “efforts to promote education” and “promotion of professional position” helped to facilitate professional growth process. They helped to promote education by using effective strategies of teaching, students’ learning concern and effort for facilitating that, effort to promote bedside and adherence to perform education rules and they helped to promote research with concern of doing research projects and using them at beside and also helped to promote professional position by attempting for professional independence, social-professional pledge and organizational-professional interactions. Some samples of participants’ experiences:

“The teacher was really dominant on the lesson and was completely prepared for the class in a way that when he/she was presenting the lesson I enjoyed”. (FG1)

“We have to check the patient holistically. Many times I say that look; now this doctor just came to look at his/her gastrointestinal. Unfortunately these things exist. It means that one person comes to just look at one system but nursing should see all and provide care”. (T5)

“It is not like this that I just want to stick to what I know, there are many studies that have been done, it is really important for me to know what did the researchers result from these studies and I use their results.” (T5)

“Their relationship with treatment staff was very respectful and what always draws my attention is that, their relationship was completely friendly and it was in the framework of professional relationships.” (FG3)

**Third phase: Evolution**

The efforts that the participants made for achieving growth and excellence lead to systematic and individual evolution. Systematic evolution was in the form of change and evolutions in clinical and educational environments and creating empowerment, freshness and vitality in educational systems. Individual positive outcomes for the students included creating professional positive attitude, motivation, training students who were evolved, promotion of educational performance, professional promotion, satisfaction, promotion of clinical practice. Among individual positive outcomes for the teachers, it can be pointed to the professor’s satisfaction and their professional success. Experiences of the participants:

“I think that the professor could transfer to us his/her interest in teaching and that he/she wanted his/her lesson to be useful for the students and the students can use it in the future, and what is interesting is that; I will say in the future that he/she taught very well and he/she influenced us, his/her practice was accepted and was considered well and he/she was as a role-model person, he was like this for me and I tried to follow him/her in different areas”. (S2)

“My students go and do a change in the unit and go and do a change in work environment and they see the effects of their programs, for example they establish a documentation system, a planning system”. (T2)

What shows the way and the process of model teachers facing with nursing students is their continuous effort for their growth and excellence; firstly it has been started by perceiving threat of inadequate individual and professional growth as the started main concern that was the factor of raising strategy in most of the participants. This effort had been started and continued in inactive and unconscious form in two basic steps (1) facilitating the professional growth (2) facilitating human development (strategies). Finally participants stated and showed growth and excellence with indexes and signs of individual and systematic change.

In line with this process they had special attention to optimal use of supportive sources (assistance of learning environment, appropriate facilities and equipment) and facilitating factors (professor’s personal and institutional power, professor’s appearance norms, student’s effort and motivation and student’s modeling) (intervening conditions) and strengthen and accelerate growth and excellence.

This process is still affected by other factors as deterrent too (busy professor, unpleasant career prospects and inappropriate conditions of educational environments) and had disruption and slowness.

**Figure 2:**
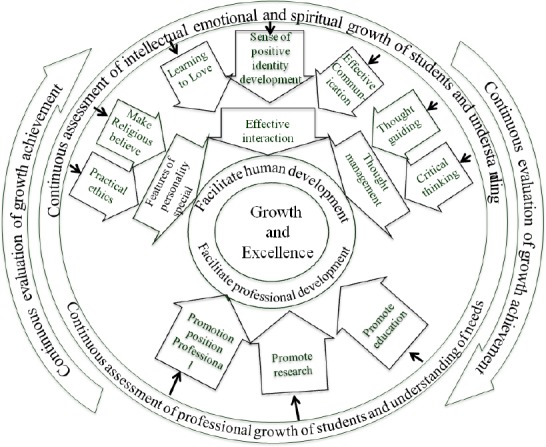
Growing up and Role Modeling Theory in nursing students’ education

According to grow up and role-modeling theory, role-modeling in teaching nursing students was a complex content, multidimensional, gradually (need to time), dynamic and progressive in line of advancing growth and excellence and it was affected by different factors (Figure 2).

## 4. Discussion

Findings showed that the dominant and the main process of facing participants was “effort for all-round growth and excellence”, actually the main mean of role-modeling for participants was stated effort for all-round growth and excellence.

Model nursing teachers by considering student’s basic needs and on the basis of perceived concerns in education environment tried for facilitating all-round growth (human and professional) of the students. Basically the aim of curriculum has been stated growth, perfection, awareness of human to nurture and develop potential talents of the students (Abdollahi, 2008). Education and training teachers should regulate and perform their curriculum, education methods and evaluation methods according to self-actualization characters and its behaviors. Transferring people’s potential abilities to personal high quality actions, attention to cognitive and emotional needs and finally achieving self-actualization, independent judgment and action, facilitating all-round growth and achieving self-evaluation should be included in educational program (Safavi, 2006).

In addition to that, growth has been mentioned as the aim of education and the teacher should help the students’ growth (Richards & Richards, 1976), among the special characteristics of role-modeling is commitment to the learners’ growth (Siegel, 2004).

In the realizing and exposure to inadequate human and professional growth phase

The threat that the participants had stated about human growth barriers (emotional, spiritual and intellectual growth) of nursing students also had been attended in other studies. As in the study of Holt-Waldo (2011) human-centered education was the concern of the teachers (Holt-Waldo, 2011; Mokhtari et al., 2012) and it has been said that human-centered class is a place where it has been trusted to the learners as the people who are self-guided, dissenting opinions and individual differences are accepted and movement towards the target is encouraged (Clark, 2008).

Although the students state that one of the rating criteria for clinical instructor is performing theoretical training in action (Heshmati-Nabavi & Vanaki, 2010) the studies which have been done in Iran show theoretical and clinical education failure for the nursing students (Ahmadinejad et al., 2002). There is high distance between nursing theory and clinical practice in nursing education and among this, nursing education system has not done well. Nursing students are dissatisfied with clinical education and they experience anxiety because of lack of achieving professional nursing knowledge and skills in taking care of the patients (Cheraghi et al., 2010); the emphasis of the model teachers about professional norms in this study should be attended, because nursing teachers have critical and fateful role in training expert and finally growth and development of the societies (Mirkamali & Narenji, 2009). They are those who have to introduce professional realities to the students (Nugent et al., 2004). Disch et al. (2004) reported that 97% of nursing faculty members is committed to their profession (Disch et al., 2004). In other studies it has been shown that clinical effective educators have special attention to professional (Salehi et al., 2004; Soltanarabshahi, & Ghaderi, 2001) and scholarship dimension (Blauvelt et al., 2011), also in this study research-centered nursing that included doing research projects, in line with educational needs and using research results in clinical and theoretical education were among concerns of nursing model teachers.

In the facilitating human and professional development phase

According to the results; model teachers’ approaches and strategies were in line with human growth (three emotional, spiritual and rational dimensions) and professional growth. Human-centered nursing education states relationship, growth, respect and look at the student with a holistic lens. Human-centered teachers develop an environment which is full with trusting on the students, so that establish healthy and encouraging relationship (Holt-Waldo, 2011). All the students need their dignity to be protected and they need to be respected (Campbell, 2006). Respecting the student is among effective teaching characteristics (Abdollahi & Heidari, 2009), so the teachers should obey clear, respectful, moral and pedagogy codes (Rama, 2011). Students in this study talked about model teachers’ effective interactions with them in all the conditions and the model teachers talked about necessity of respecting the students and adhering to that.

Model teachers of this study following relationship with God and religious beliefs as a Muslim had mental health, practical ethics and its consequents, it means; sincerity, honesty, patience, soberity, good temper, regulation, accepting criticism, responsibility (work commitment), firmness and seriousness, humility, dedication, endurance and sustainability, flexibility, confidence, eagerness and ability to love and they caused spiritual growth of the students. These characteristics of the model teachers are among expected characteristics of an effective teacher who is the favorite teacher of the students (Abdollahi, 2008).

Teaching critical thinking by designing curious questions (Rama, 2011) is known as the most important efforts which have to be made in teaching nursing students (Vaghar Seyyedin A et al., 2009). Providing role-model behaviors has been mentioned as the cause of encouraging critical thinking in students (Williams-Barnard et al., 2006). Strengthening active and critical thinking by making thinking challenges, creating chat room, designing questions and answers, forcing students to think and strengthening criticism power had been facilitated by model teachers.

Attending development and professional growth of nursing students who are future of this profession were also considered by nursing model teachers. Role-modeling is the heart of being professional (Kenny et al., 2003). Role-modeling is the accepted strategy for transferring professional attitudes and behaviors from nursing teachers to the students (Bidwell & Brasler, 1989; Perry, 2009).

Facilitating learning is the main effort of model teachers for promoting education in the students. It has been done through consultation operational planning and continuous guidance. It is also in the studies that humanistic teachers are the facilitators of learning (Holt-Waldo, 2011). Verst (2010) writes that the teachers must create an environment that facilitates students’ learning (Verst, 2010), facilitating learning is nursing teacher’s duty (Farmer & Frenn, 2009) and it has been introduced as one of the main qualifications of nursing teachers (Blauvelt et al., 2011). Among the main qualifications of nursing teachers is involving in scholarship (Blauvelt et al., 2011). Also in this study research-centered nursing that included doing research projects in line with educational needs and using research results in theoretical and clinical teaching were among concerns of nursing model teachers. Successful model teachers had the concern of promoting care method by the students at bedside that they showed this concern by applying theory in practice, involvement in providing care, attending to holistic and human-centered care and doing care eagerly. Role-modeling supports combination of theory and bedside and it helps to promote profession in the future (Perry, 2009).

Another finding was model teachers’ use of effective evaluation methods. About the importance of evaluation, we point to some of the studies: one of the main challenges of nursing education is evaluation of clinical qualification because evaluation can show students’ abilities and their required resources (McWilliam & Botwinski, 2010).

In order to maintain the integrity of nursing profession, it is necessary to promote nursing discipline and profession (Krause, 1993). Also in this study model teachers believed in obeying law and the rule of law). What has been found in this study was attending interprofessional interactions and interprofessional relationships in model teachers; also studies stated interprofessional participation and interprofessional mentoring approach and they introduced that as an effective learning strategy for increasing students’ knowledge and skills in interprofessional skills (Lait et al., 2011). So successful model teachers helped to promote the profession by appropriate interprofessional interactions.

In the evolution phase

Model teachers made individual and systematic changes, it has been pointed out in other studies that the top teachers have positive effect on their students’ future (Verst, 2010), models act as a catalyst for transmission (evolution) (Perry, 2009). Stated feel of being supported by the students from the model teachers can help the students’ self-efficacy (Rostami et al., 2010) and increase of their general health (Peyravi et al., 2010). Result of effective interactions of model teachers with students indicate creating motivation for accepting responsibility of learning, which is teachers’ duty for effective learning (Panduragan et al., 2011; Verst, 2010). Top teachers create motivation for the students to accept responsibility of their learning (Verst, 2010).

Mastery of knowledge makes power (Brijnath & Manderson, 2008) and in this study model teachers because of the superiority in knowledge had the power of making individual change in the students and work environments. Also there was feeling of power in their students because of master in science. On the other hand model teachers by facilitating emotional growth caused instilling sense of positive identity in the students (Aston & Molassiotis, 2003). Also ethical behaviors of nursing model teachers can cause ethics nurturing in the nursing students (Duchscher, 2000). Nurturing scientific power, sense of positive identity and ethics in the students by model teachers were among predisposing factors for making systematic evolution in work environment by the students.

Among other concerns of model teachers were professional independence and showing nursing profession scientific identity and the studies show that professional growth and independence cause job satisfaction (Gui et al., 2009), recruitment and retention of nursing teachers and it is going to be totally effective in nursing education (Grandjean et al., 1976).

## 5. Implications for Nursing & Health Policy

Accordingly, the design and implementation of the designed model can be used to make this unconscious to conscious, active and voluntarily process; a process to help nursing faculties education administrators and supra organization to prevent threats to human and nursing students’ professional education and promotion of nursing students’ growth.

## 6. Conclusion

The role modeling process is taking place as an unconscious, involuntary, dynamic and positive progressive process in order to facilitate overall growth in nursing students. Accordingly, implementation of the designed Grow up and Role Modeling Theory can be used to make this unconscious to conscious, active and voluntarily process; a process to help nursing schools education administrators and supra organization to prevent threats to human and nursing students’ professional education and facilitate nursing students’ growth. It is hoped that this model to be tested in comprehensive and based on students’ needs to intervention studies and leads to desired interpersonal and systemic consequences in the education of nursing students.
